# Assessment of a Nutritional Rehabilitation Model in Two Modern Broilers and Their Jungle Fowl Ancestor: A Model for Better Understanding Childhood Undernutrition

**DOI:** 10.3389/fnut.2018.00018

**Published:** 2018-03-23

**Authors:** Mikayla F. A. Baxter, Juan D. Latorre, Dawn A. Koltes, Sami Dridi, Elizabeth S. Greene, Stephen W. Bickler, Jae H. Kim, Ruben Merino-Guzman, Xochitl Hernandez-Velasco, Nicholas B. Anthony, Walter G. Bottje, Billy M. Hargis, Guillermo Tellez

**Affiliations:** ^1^Department of Poultry Science, University of Arkansas, Fayetteville, AR, United States; ^2^Department of Animal Science, Iowa State University, Ames, IA, United States; ^3^Department of Pediatrics, University of California, San Diego, San Diego, CA, United States; ^4^Division Neonatology, University of California, San Diego, San Diego, CA, United States; ^5^College of Veterinary Medicine, National Autonomous University of Mexico, Ciudad de Mexico, Mexico

**Keywords:** nutritional rehabilitation, chicken lines, compensatory growth, bone mineralization, morphometric analysis

## Abstract

This article is the first in a series of manuscripts to evaluate nutritional rehabilitation in chickens as a model to study interventions in children malnutrition (Part 1: Performance, Bone Mineralization, and Intestinal Morphometric Analysis). Inclusion of rye in poultry diets induces a nutritional deficit that leads to increased bacterial translocation, intestinal viscosity, and decreased bone mineralization. However, it is unclear the effect of diet on developmental stage or genetic strain. Therefore, the objective was to determine the effects of a rye diet during either the early or late phase of development on performance, bone mineralization, and intestinal morphology across three diverse genetic backgrounds. Modern 2015 (Cobb 500) broiler chicken, 1995 Cobb broiler chicken, and the Giant Jungle Fowl were randomly allocated into four different dietary treatments. Dietary treatments were (1) a control corn-based diet throughout the trial (corn–corn); (2) an early phase malnutrition diet where chicks received a rye-based diet for 10 days, and then switched to the control diet (rye–corn); (3) a malnutrition rye-diet that was fed throughout the trial (rye–rye); and (4) a late phase malnutrition diet where chicks received the control diet for 10 days, and then switched to the rye diet for the last phase (corn–rye). At 10 days of age, chicks were weighed and diets were switched in groups 2 and 4. At day 20 of age, all chickens were weighed and euthanized to collect bone and intestinal samples. Body weight, weight gain, and bone mineralization were different across diet, genetic line, age and all two- and three-way interactions (*P* < 0.05). Overall, Jungle Fowl were the most tolerant to a rye-based diet, and both the modern and 1995 broilers were significantly affected by the high rye-based diet. However, the 1995 broilers consuming the rye-based diet appeared to experience more permanent effects when compared with the modern broiler. The results of this study suggest that chickens have a great potential as a nutritional rehabilitation model in human trials. The 1995 broilers line was an intermediate genetic line between the fast growing modern line and the non-selected Jungle Fowl line, suggesting that it would be the most appropriate model to study for future studies.

## Introduction

Malnutrition is a growing concern as the global population continues to increase due to the increased global demand for food and the long-term effects of malnutrition. Malnutrition is due to the lack of, inadequate nutrition, or inadequate absorption of nutrients. It has been identified as the underlying cause of death in one-third of children under 5 years of age. Of these deaths, 83% are attributed to mild-to-moderate malnutrition compared with severe malnutrition (1), suggesting that while malnutrition plays a major role in child mortality current strategies involving only the treatment of the severely malnourished may not be enough to reduce the negative impacts of malnutrition (2–4).

The most common manifestation of chronic malnutrition is stunted growth. It is estimated to affect 165 million children under the age of 5 years in low- and middle-income countries (5). The critical period when stunting can develop is between pregnancy and the first 2 years of life (the first 1,000 days) (6, 7) and can be temporary or permanent. If the nutrient restriction is severe enough, permanent stunted growth may occur (1). While stunting is an outward consequence malnutrition, early life malnutrition may adversely affect brain anatomy, physiology, biochemistry, or lead to permanent brain damage (8). Currently, strategies to prevent malnutrition focus on providing proper nutrition to overcome the condition, but research has rarely examined the consequences of these feeding strategies which may be due to limited availability of models.

Corn is the main energy source in poultry diets. However, it can be cost prohibitive to include in the diet at times. Unconventional grain sources can be used to reduce or replace corn usage during times that are cost prohibitive. Unlike corn, rye contains of a high amount of non-starch polysaccharides (NSP), which impairs nutrient digestion and absorption due to little or no intrinsic enzymes capable of hydrolysis of NSP in the small intestine. The increased NSP provide more nutrients in the ceca and large intestine which serve as a nutrient source for bacteria. The altered nutrient source can lead to dysbiosis within the gut. In poultry, rye-based diets increased both viscosity and *Clostridium perfringens* and *Clostridium difficile* proliferation when compared with corn-based diets (9). Additionally due to the anti-nutritive properties of rye, poultry consuming rye diets experience stunting and many similar pathologies associated with malnutrition in children including development of enteric enteropathy, alteration in gut microbiome profile, bacterial translocation, reduction in nutrient digestion and absorption, as well as poor bone mineralization (4, 10–17). These similarities between chickens consuming rye diets and malnourished children may make poultry a potentially good model to understand short- and long-term effects of malnourishment; however, it is unclear how selection practices in the broiler industry may alter these effects.

Undernutrition of children has profound effects on health and development; nevertheless, the issue is not simply caused by a lack of food, but results from complex interactions of intra- and intergenerational factors (4, 18). Research on human nutrition has relied heavily on animal models for its insights (19, 20). Avian models, specifically in the chicken, have been essential in contributing to the current understanding of several nutrient deficiencies, nutrient interactions, bioavailability, digestibility, tolerances, and toxicities (21–23). Basic mechanisms of the enteric nervous system, the gut-associated lymphoid tissue, and intestinal permeability are highly conserved across animal species. However, there are gastrointestinal physiological similarities between chickens and humans that make chickens a viable nutritional model when studying human nutrition: both species lipogenesis primarily takes place in the liver, iron is absorbed in the duodenum and neonatal humans, and chickens can utilize sucrose as energy source (24–27). Finally, in contrast with other animal models, chickens consuming diets high in NSP developed severe gut inflammation, accompanied with dysbacteriosis, decreased nutrient absorption, poor bone mineralization, and increased liver bacterial translocation (15, 28). Some of these clinical signs are similar to what patients with environmental enteropathy (EE) experience. EE is an enigmatic disorder that often occurs in young children living in unsanitary conditions (12, 17). Also, EE is characterized by reduced intestinal absorptive capacity, altered gut barrier function, intestinal inflammation, and dysbacteriosis (13, 14). Therefore, patients with EE and chicks consuming diets high in NSP develop similar physiopathology (15, 16), making chickens a viable model when determining the effects of diet on childhood malnutrition.

Following nutrition deprivation, many organisms can undergo accelerated growth to return to a normal weight range, or also referred to compensatory growth (18, 29–31). In some instances, body weight (BW) of animals under feed restriction will catch-up to control animals with *ad libitum* feed intake (18, 30). In fact, high compensatory growth rates in feed restriction animals result in overcompensation due to excessive fat deposition and animals recover to normal weight without additional time (29, 31). Nevertheless, when the nutrient restriction is severe, the growth period must be extended to reach the normal weight, but if the nutrient restriction is severe enough, permanent stunted growth may occur (1). Some of the factors that affect compensatory growth include composition of the restricted diet, severity of undernutrition, duration of the period of undernutrition, age, genotype, and gender among others (32–35). Therefore, understanding the effects of compensatory growth following nutritional deficiencies could allow for strategies to be developed to mitigate the long-term effects of early childhood malnutrition.

Genetic selection has made modern broilers a unique model for understanding growth. It has allowed broiler chickens to double their starting BW in 3 days, and reach puberty in 4.5 months (36, 37). In addition, extensive work has been done to determine optimal nutrition and management for growth due to the increased pressures to improve and maintain high efficiency in agriculture production. In addition to modern poultry lines, university have preserved minimally or unselected poultry lines. The Red Jungle Fowl is the closest living ancestor to the modern chicken and can be considered as a “wild type” in poultry genomics (38). Previous research determined that Jungle Fowl took 93 days longer to reach the same physiological BW than broiler breeders and had significantly lower average daily gain (38). As well, the University of Arkansas maintains a random bred control line, which is the product of intercrossing 13 commercial broilers, parent lines from 1997 (39). This unique set-up allows for researcher to determine the effects of slight or severe malnutrition and nutritional recovery on performance and physiology. Therefore, we wanted to determine the effects of a malabsorptive diet during an early or a late growth phase on growth and bone and intestinal development across diverse genetic backgrounds. For this study, we utilized a rye diet which has been shown to induce nutritional deficiencies (15, 40–43) when compared with a control corn diet across a modern commercial broiler, a commercial broiler of 1995 genetics, and an unselected Jungle Fowl line.

## Materials and Methods

### Animal Source, Diets, and Experimental Design

**Figure 1:**
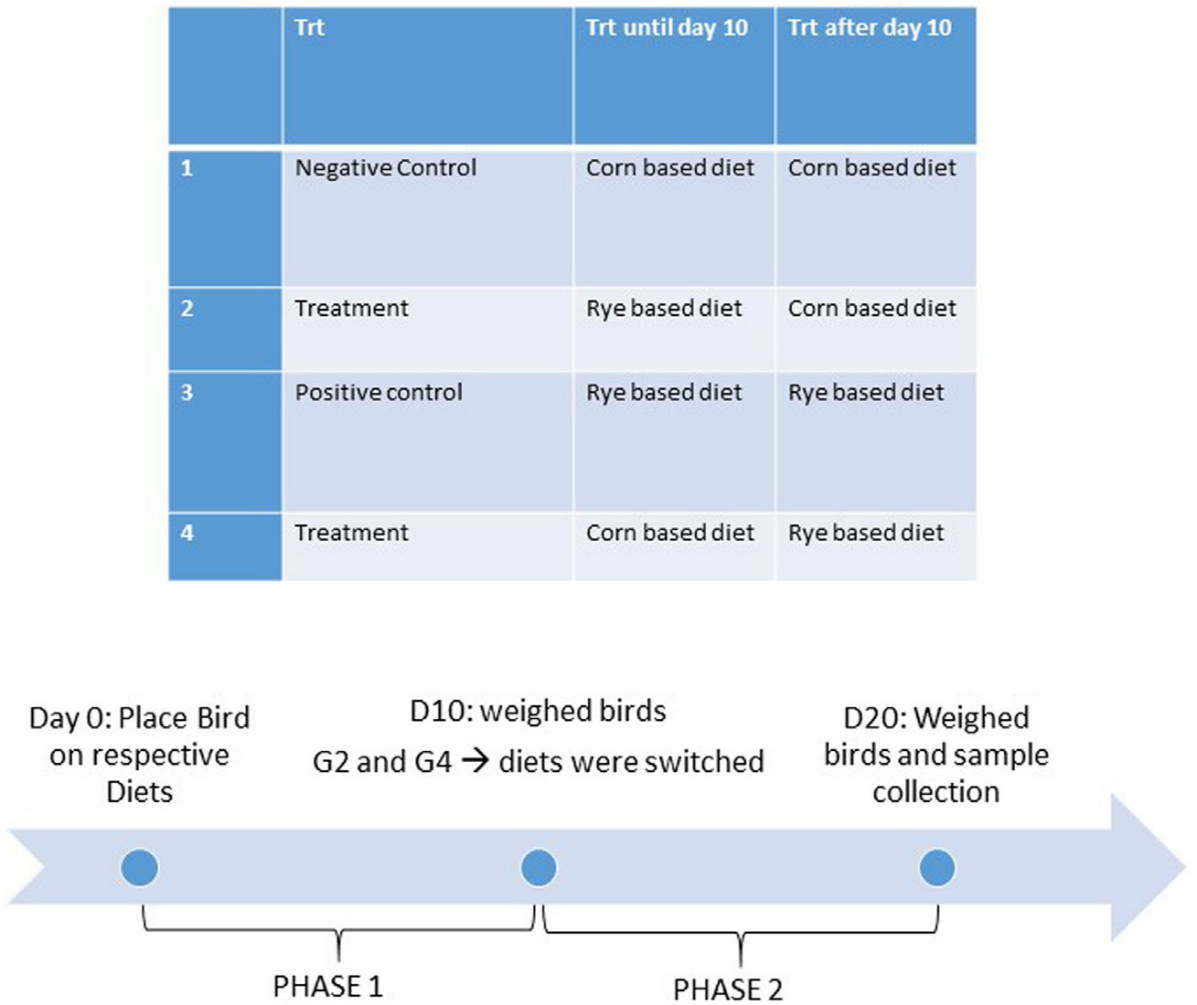
Dietary treatments and timeline.

All animal procedures were approved and in compliance with Institutional Animal Care and Use Committee at the University of Arkansas, Fayetteville (protocol #15006). The three lines of chickens included in this study. For the modern broiler chickens, 160 1-day-old mixed broiler chicks, Cobb-Vantress, Silom Springs, AR, USA were used (*n* = 40 chickens/group). For the 1995 broiler chickens, 112 1-day-old mixed broiler chicks, from the random bred line initiated from 1995 Cobb broiler chicken line (39) were used (*n* = 28 chickens/group). And for the Jungle Fowl chickens, 160 1-day-old mixed Giant Jungle Fowl (44) were used (*n* = 40 chickens/group). On the day of hatch, chickens were neck-tagged, weighed, and randomly allocated to one of four dietary treatment groups in floor pens containing new pine shavings in an environmentally controlled room. All diets were antibiotic-free and formulated to meet or exceed the current broiler nutritional requirements according to the National Research Council [Ref. (45); Table 1]. When administering dietary treatment, the experiment was split into two phases, the first phase was from day of hatch to day 10 and the second phase was from day 10 to day 20. Dietary treatments were (1) a control diet where chicks were maintained on a corn-based diet throughout the trial (corn–corn); (2) an early phase malnutrition diet where chicks were on a rye-based diet for 10 days, and then switched to the control diet (rye–corn); (3) a malnutrition rye-diet that was fed throughout the trial (rye–rye); and (4) a late phase malnutrition diet where chicks received the control diet for 10 days, and then switched the rye diet for the last phase (corn–rye) (Figure 1). Temperature was maintained according to normal management practices (34°C for the first 5 days then gradually reduced to 23°C). Individual BW were recorded at day of hatch, day 10 and day 20. Body weight gain (BWG) was calculated by subtracting the initial BW from the final BW and was calculated from day of hatch to day 10 and from day 10 to day 20. Chickens were euthanized *via* carbon dioxide asphyxiation and samples were collected for bone and intestinal measurements on day 20.

**Table 1:** Composition and nutrient content of the experimental diets (%).

Item Ingredients (%)	Rye-based diet	Corn-based diet
Corn	–	57.32
Rye	58.27	–
Soybean meal	31.16	34.66
Poultry fat	6.30	3.45
Dicalcium phosphate	1.80	1.86
Calcium carbonate	1.10	0.99
Salt	0.38	0.38
d,l-Methionine	0.35	0.33
Vitamin premix^a^	0.10	0.20
l-Lysine HCl	0.22	0.31
Choline chloride 60%	0.10	0.20
Mineral premix^b^	0.12	0.12
Threonine	0.08	0.16
Antioxidant^c^	0.02	0.02
**Calculated analysis**		
Metabolizable energy (kcal/kg)	2,850	3,035
Crude protein, %	22.38	22.16
Lysine, %	1.32	1.35
Methionine, %	0.64	0.64
Methionine + cysteine, %	0.98	0.99
Threonine, %	0.86	0.91
Tryptophan, %	0.30	0.28
Total calcium, %	0.90	0.9
Available phosphorus (%)	0.45	0.45
Sodium (%)	0.16	0.16

*^a^Vitamin premix supplied the following per kg: vitamin A, 20,000 IU; vitamin D3, 6,000 IU; vitamin E, 75 IU; vitamin K3, 6.0 mg; thiamine, 3.0 mg; riboflavin, 8.0 mg; pantothenic acid, 18 mg; niacin, 60 mg; pyridoxine, 5 mg; folic acid, 2 mg; biotin, 0.2 mg; cyanocobalamin, 16 µg; and ascorbic acid, 200 mg (Nutra Blend LLC, Neosho, MO 64850)*.

*^b^Mineral premix supplied the following per kg: manganese, 120 mg; zinc, 100 mg; iron, 120 mg; copper, 10–15 mg; iodine, 0.7 mg; selenium, 0.4 mg; and cobalt, 0.2 mg (Nutra Blend LLC, Neosho, MO 64850)*.

*^c^Ethoxyquin*.

### Bone Parameters

Tissue was removed from the both the left and right tibias from each chicken (*n* = 10/group). Bone ash was measured on the left tibia according to published methods (46). Briefly, bones were dried at 100°C for 24 h and weighed, then ashed in a muffle furnace (Isotemp muffle furnace, Fisher Scientific, Pittsburgh, PA, USA) at 600°C for 24 h, cooled in a desiccator, and weighed. Bone mineral analysis was conducted for bone calcium and phosphorus content in the left tibia as well, using standard AOAC guidelines (47). For breaking strength, the right tibial diaphyses were cleaned of adherent tissues, the periosteum removed, and the biomechanical strength of each bone was measured using an Instron 4502 (Norwood, MA, USA) with a 509 kg load cell using recommended protocols. Bones were held in identical positions. The mid-diaphyseal diameter of the bone at the site of impact was measured using a dial caliper. The maximum load at failure was determined using a three-point flexural bend fixture with a total distance of 30 mm between the two lower supporting ends. The load, defined as force in kg per mm^2^ of cross-sectional area (kg/mm^2^). We will refer to this as bone strength. The rate of loading was kept constant at 20 mm/min collecting 10 data points per second using the Instron’s Series IX Software (Norwood, MA, USA).

### Histology and Morphometric Analysis of the Intestine

Intestinal sections (0.5 cm) were collected from the middle of the descending duodenal loop, and at Meckel’s diverticulum. These sections will be referred to as the duodenum and ileum, respectively. Tissue was fixed in 10% neutral-buffered formalin, embedded in paraffin, sectioned (5-µm thick), and stained with hematoxylin and eosin, then examined by light microscopy. Photomicrographs of random chosen fields of each intestinal section were acquired using a microscope equipped with a Leica DFC450C camera and Leica V 3.8.0. software (Leica Application Suit). ImageJ 1.47v software was used for the morphometric analysis of villus height (VH), crypt depth (CD), and muscularis width (48). For each sample, 10 measurements were taken. Measurements from the VH and CD were used to calculate the villus height to crypt depth (VH:CD) ratio.

### Statistical Analysis

Data were analyzed using a linear mixed model procedure in SAS [PROC MIXED (49)] where the factors of dietary treatment, genetic line, and the interaction between the dietary treatment and genetic line were fit as fixed effects for all variables. Since birds were weighed multiple times, a repeated effect for day was fit when analyzing BW and BWG data, and age of the bird along with the two- and three-way interactions were included at fixed effects in the statistical model. Significant was set at a *P* < 0.05. When factors were determined to be significant, pairwise comparisons were performed in SAS using the LSmean statement and corrected for multiple tests using Tukey’s *post hoc* adjustment.

## Results

### Body Weight

The results of the evaluation of a nutritional rehabilitation model on BW in three genetic chicken lines fed rye or corn at varying time points are summarized in Table 2. As to be expected, BW increased as birds aged (*P* < 0.0001) regardless of dietary treatment and genetic line. There was a significant difference in BW between all treatment groups, where the corn–corn group (*P* < 0.0001) had the highest BW, and the rye–rye group (*P* < 0.0001) had the lowest BW. Modern broilers were the heaviest, followed by the 1995 broilers and then the Jungle Fowl (*P* < 0.0001).

**Table 2:** Evaluation of a nutritional rehabilitation model on body weight in three genetic chicken lines fed rye or corn at varying time points.

Day	Treatment	Genetic line			Variable	*P*-value
		Modern broiler	1995 Line	Jungle Fowl		
1	Corn	40.11 ± 0.33^a,z,3^	40.75 ± 0.39^a,z,3^	34.26 ± 0.33^a,z,3^	trt	<0.0001
	Rye	39.85 ± 0.33^a,z,3^	40.37 ± 0.40^a,z,3^	33.65 ± 0.33^a,z,3^	line	<0.0001
10	Corn	175.91 ± 1.73^a,x,2^	123.71 ± 2.07^a,y,2^	74.45 ± 1.73^a,z,2^	day	<0.0001
	Rye	151.74 ± 1.76^b,x,2^	95.3 ± 2.11^b,y,2^	69.08 ± 1.77^a,z,2^	trt*line	<0.0001
20	Corn–corn	715.5 ± 5.84^a,x,1^	528.07 ± 6.75^a,y,1^	190.05 ± 5.84^a,z,1^	trt*day	<0.0001
	Rye–corn	695.85 ± 5.84^a,x,1^	340 ± 6.75^b,y,1^	165.4 ± 5.84^a,b,z,1^	line*day	<0.0001
	Rye–rye	393.59 ± 6.34^c,x,1^	231.2 ± 6.75^d,y,1^	143.45 ± 5.84^b,z,1^	trt*line*day	<0.0001
	Corn–rye	453.8 ± 5.84^b,x,1^	280.88 ± 6.53^c,y,1^	173.4 ± 5.84^a,b,z,1^		

*Data are expressed as the LSmean ± SE*.

*^a–c^Significant differences between the treatments within the column at each time point*.

*^x–z^Significant difference between the genetic lines within the rows at each time point*.

*^1–3^Significant difference between the collection days within the columns at each time point*.

Regardless of dietary treatment, there was a significant increase in BW from day 1 to day 10 and day 10 to day 20 in the Jungle Fowl, 1995 broilers, and the modern broiler. Overall, there was a significant dietary treatment by genetic lines interaction (*P* < 0.0001), where modern broilers in the corn–corn group were heaviest (*P* < 0.0001) and Jungle Fowl in the rye–rye group were the lightest (*P* < 0.0001). Across genetic line at both day 10 and day 20, the modern broiler were heavier than the 1995 broiler or the Jungle Fowl for all treatment groups (*P* < 0.0001). Also, 1995 boilers weighed more than the Jungle Fowl for all treatments groups (*P* < 0.0001). Genetic line had a significant effect on BW at 9 days of age where regardless of diet, modern broilers weighed more than the 1995 broilers and Jungle Fowl (*P* < 0.0001) and 1995 broilers weighed more than Jungle Fowl (*P* < 0.0001).

At day 10, modern broiler in the rye–rye treatment group had significantly lower BW than the corn–corn (*P* = 0.0016) and corn–rye (*P* = 0.0106). There was no difference in BW between treatment groups consuming the same diet (corn–corn vs corn–rye and rye–rye vs rye–corn; *P* = 1.00). Surprisingly, there was also no difference in BW between rye–corn treatment group and the corn–corn (*P* = 0.2104) and the corn–rye (*P* = 0.2979) treatment groups. However, during the first phase of the experiment, chicks in the corn–corn and corn–rye were both consuming a corn-based diet, just as chicks on the rye–rye and rye–corn groups were both consuming a rye-based diet. Therefore, when BW of chicks consuming the same diets were combined (corn–corn with corn–rye and the rye–rye with rye–corn), corn-fed chicks weighed significantly more than rye-fed chicks (*P* < 0.0001; Table 2). At day 10, 1995 broilers in the rye–corn treatment group had significantly lower BW than the corn–corn (*P* = 0.0002) and corn–rye (*P* = 0.0008). There was no difference in BW between the corn–corn and corn–rye treatment groups (*P* = 1.000) nor was there a difference in BW between the rye–rye and rye–corn treatment groups (*P* = 0.8282). Similar to what was observed in the modern broilers, there was also no difference in BW between rye–rye treatment group and the corn–corn (*P* = 0.412) and the corn–rye (*P* = 0.6993) treatment groups. However, when BW of chicks consuming the same diets were combined (corn–corn with corn–rye and the rye–rye with rye–corn), corn-fed chicks weighed significantly more than rye-fed chicks (*P* < 0.0001; Table 2). There was no difference in BW between treatments in Jungle Fowl at day 10.

At day 20, modern broiler in the corn–corn and rye–corn groups did not differ in BW (*P* = 0.8935) but weighed significantly more than those fed rye in the second phase of the experiments (*P* < 0.0001). Also, modern broilers in the corn–rye group weigh significantly more than the rye–rye fed chicks (*P* < 0.0001). BW of 1995 broilers at 20 days of age was significantly different between all treatment groups, where the corn–corn group weighed significantly more than those fed rye at any phase of the experiment (*P* < 0.0001). The rye–corn group weight significantly more than the rye–corn and the rye–rye group (*P* < 0.0001). 1995 Broiler in the rye–rye group had significantly lower BW than the corn–rye group (*P* = 0.0007). At day 20, Jungle Fowl in the corn–corn group weigh significantly more than the rye–rye group (*P* = 0.0002); however, there were no significant difference in BW between the other treatments. Within each dietary treatment, there was a significant difference in BW, where modern broilers weighed more than both 1995 broilers and Jungle Fowl (*P* < 0.0001), and 1995 broiler weighed more than Jungle Fowl (*P* < 0.0001).

### Body Weight Gain

Table 3 shows the evaluation of a nutritional rehabilitation model on average BWG in three genetic chicken lines fed rye or corn at varying time points. As expected, BWG increased over time where BWG from day 10 to day 20 was significantly higher than BWG from day 1 to day 10 (*P* < 0.0001). Dietary treatment had a significant effect on BWG (*P* < 0.0001), regardless of day or genetic line, where chicks in the corn–corn group had significantly higher BWG than those fed rye at any phase of the experiment (*P* < 0.0001). The rye–corn group had the second highest BWG which was significantly higher than those chicks fed rye in the second phase of the experiment (*P* < 0.0001). Also, the corn–rye group had a significantly higher BWG than the rye–rye group (*P* < 0.0001). Genetic line had a significant effect on BWG, where modern broilers had significantly higher BWG than the 1995 broiler (*P* < 0.0001) and Jungle Fowl (*P* < 0.0001). As well, 1995 broiler had significantly higher BWG than the Jungle Fowl (*P* < 0.0001).

**Table 3:** Evaluation of a nutritional rehabilitation model on average body weight gain in three genetic chicken lines fed rye or corn at varying time points.

Day	Treatment	Genetic line			Variable	*P*-value
		Modern broiler	1995 Line	Jungle Fowl		
1–10	Corn	135.80 ± 1.78^a,x,2^	81.46 ± 2.12^a,y,2^	40.19 ± 1.78^a,z,2^	trt	<0.0001
	Rye	112.63 ± 1.79^b,x,2^	53.36 ± 2.12^b,y,2^	34.81 ± 1.79^a,z,2^	line	<0.0001
					day	<0.0001
10–20	Corn–corn	546.4 ± 6.80^a,x,1^	384.93 ± 7.85^a,y,1^	116.15 ± 6.80^a,z,1^	trt*line	<0.0001
	Rye–corn	534.40 ± 6.80^a,x,1^	248.87 ± 7.85^b,y,1^	98.35 ± 6.80^a,b,z,1^	trt*day	<0.0001
	Rye–rye	251.29 ± 7.37^b,x,1^	117.20 ± 6.80^d,y,1^	74.85 ± 6.80^b,z,1^	line*day	<0.0001
	Corn–rye	278.50 ± 6.80^b,x,1^	178.62 ± 7.60^c,y,1^	95.70 ± 6.80^a,b,z,1^	trt*line*day	<0.0001

*Data are expressed as the LSmean ± SE*.

*^a–c^Significant differences between the treatments within the column at each time point*.

*^x–z^Significant difference between the genetic lines within the rows at each time point*.

*^1–2^Significant difference between the collection days within the columns at each time point*.

Interaction between the age of the chicks, dietary treatment and genetic line had a significant effect on the BWG (*P* < 0.0001). At day 0–10, modern broilers in the rye–corn treatment group had significantly lower BWG than the corn–corn (*P* = 0.0011) and corn–rye (*P* = 0.0033). BWG from day 1 to day 10 in modern broilers was significantly lower in the rye–rye treatment group when compared with the corn–corn (*P* = 0.0233) and corn–rye (*P* = 0.0205). There was no difference in BWG between treatment groups consuming the same diet (corn–corn vs corn–rye and rye–rye vs rye–corn; *P* = 1.00), which was expected. There was also no difference in BWG between rye–corn treatment group and the corn–corn (*P* = 0.4095) and the corn–rye (*P* = 0.3831) treatment groups. However, when BWG of chicks consuming the same diets were combined for day 1–10 (corn–corn with corn–rye and rye–rye with rye–corn), corn-fed chicks had a significantly higher BWG than rye-fed chicks (*P* < 0.0001; Table 3). At day 0–10, 1995 broilers in the rye–corn treatment group had significantly lower BWG than the corn–corn (*P* = 0.0011) and corn–rye (*P* = 0.0033). There was no difference in BWG between the corn–corn and corn–rye treatment groups (*P* = 1.000) nor was there any difference in BWG between the rye–rye and rye–corn (*P* = 0.844). However, there was also no difference in BWG between rye–rye treatment group and the corn–corn (*P* = 0.6934) and the corn–rye (*P* = 0.8645) treatment groups. When BWG of chicks consuming the same diets were combined (corn–corn with corn–rye and the rye–rye with rye–corn), corn-fed chicks weighed significantly more than rye-fed chicks (*P* < 0.0001; Table 3). BWG from day 1 to day 10 was not statistically different between dietary treatment groups in Jungle Fowl. Between genetic lines, modern broilers fed corn or rye had significantly higher BWG from day 1 to day 10 than the 1995 broiler (*P* < 0.0001) and Jungle Fowl (*P* < 0.0001). As well, 1995 broilers fed corn in the first phase of the experiment had significantly higher BWG from day 1 to day 10 than Jungle Fowl (*P* < 0.0001).

From day 10 to day 20 in modern broilers, there was no significant difference in BWG between the corn–corn and rye–corn treatment groups (*P* = 0.9999) nor was there a statistical difference between the rye–rye and corn–rye groups (*P* = 0.5103). Modern broilers in the corn–corn and rye–corn groups had significantly higher BWG than the rye–rye and corn–rye groups (*P* < 0.0001). BWG from day 10 to day 20 in the 1995 broiler line was statistically different between all treatment groups, where the corn–corn group had the highest BWG (*P* < 0.0001), followed by the rye–corn group (*P* < 0.0001), then the corn–rye group (*P* < 0.0001), and lastly the lowest BWG occurred in the rye–rye group (*P* < 0.0001). From day 10 to day 20, Jungle Fowl in the corn–corn group had significantly higher BWG than the rye–rye treatment group (*P* = 0.0059); however, there were no difference in BWG between any of the other treatment groups. There was significant differences in BWG between genetic lines in the corn–corn and rye–corn treatment groups from day 10 to day 20, where the modern broilers had the highest BWG (*P* < 0.0001), followed by the 1995 broiler (*P* < 0.0001), and Jungle Fowl had the lowest BWG (*P* < 0.0001). A similar trend was observed in the corn–rye groups, where modern broilers in the corn–rye group had the highest BWG (*P* < 0.0001), followed by the 1995 broiler (*P* < 0.0001), with the Jungle Fowl having the lowest BWG (*P* < 0.0001). In the rye–rye group, modern broilers had significantly higher BWG than both the 1995 broiler and the Jungle Fowl (*P* < 0.0001). 1995 Broiler in the rye–rye treatment group also had significantly higher BWG than the Jungle Fowl (*P* = 0.0039).

### Bone Parameters

#### Tibia Strength

Table 4 summarizes the bone parameters of three different genetic lines of chickens lines fed rye or corn at varying time points at 20 days of age. Tibia strength between treatments, genetics lines and interaction between the two variables were statistically different (*P* < 0.001). Modern chickens fed corn in the second phase of the experiment had significantly higher tibia strength when compared with rye-fed chickens (*P* < 0.0001). In the 1995 chicks, the corn–corn group had significantly higher tibia strength than those chicks fed rye in any phase of the experiment (*P* < 0.0001). Chicks in the rye–corn group had significantly stronger tibias than the rye–rye (*P* < 0.0001) and the corn–rye (*P* = 0.0065) groups; however, there were no difference in tibia strength between the rye–rye and corn–rye groups (*P* = 0.9511). There were no significant differences in tibia strength between treatments in the Jungle Fowl. When comparing genetic lines within each treatment group, the modern broilers and 1995 broilers had significantly stronger tibias than the Jungle Fowl in the corn–corn group (*P* < 0.0001). Modern broilers had significantly stronger tibias than the 1995 broilers in the rye–rye (*P* = 0.0465) and corn–rye group (*P* = 0.0153). Finally, in the rye–corn group, modern broilers had significantly stronger tibias than the 1995 broilers and Jungle Fowl (*P* < 0.0001).

**Table 4:** Evaluation of a nutritional rehabilitation model on bone parameters in three genetic chicken lines fed rye or corn at varying time points at day 20 of age.

Treatment/variable	Genetic line			Variable	*P*-value
	Modern broiler	1995 Line	Jungle Fowl		
**Tibia strength (kg/mm^2^)**					
Corn–corn	3.90 ± 0.12^a,y^	3.47 ± 0.14^a,y^	1.90 ± 0.15^a,z^	trt	0.0001
Rye–corn	3.73 ± 0.15^a,y^	2.18 ± 0.16^b,z^	1.53 ± 0.15^a,z^	line	0.0001
Rye–rye	1.71 ± 0.14^b,y^	1.06 ± 0.14^c,z^	1.28 ± 0.15^a,y,z^	trt*line	0.0001
Corn–rye	2.04 ± 0.13^b,y^	1.34 ± 0.14^c,z^	1.46 ± 0.15^a,y,z^		
**Tibia ash (%)**					
Corn–corn	55.79 ± 0.79^a,x^	51.35 ± 0.88^a,y^	46.81 ± 0.95^a,z^	trt	<0.0001
Rye–corn	54.26 ± 0.88^a,y^	47.43 ± 1.02^a,z^	44.17 ± 0.95^a,z^	line	<0.0001
Rye–rye	45.76 ± 0.88^b,y^	37.19 ± 1.02^b,z^	42.31 ± 0.95^a,y^	trt*line	<0.0001
Corn–rye	46.30 ± 0.83^b,y^	38.11 ± 0.88^b,z^	43.87 ± 0.80^a,y^		
**Tibia calcium (ppm)**					
Corn–corn	41.23 ± 0.37^a,y^	38.92 ± 0.42^a,z^	39.28 ± 0.44^a,z^	trt	<0.0001
Rye–corn	41.94 ± 0.42^a,y^	38.24 ± 0.48^a,b,z^	39.40 ± 0.44^a,z^	line	<0.0001
Rye–rye	39.13 ± 0.42^b,z^	37.80 ± 0.42^a,b,z^	38.50 ± 0.44^a,z^	trt*line	0.0018
Corn–rye	38.26 ± 0.39^b,y,z^	36.42 ± 0.42^b,z^	39.05 ± 0.44^a,y^		
**Tibia phosphorus (ppm)**					
Corn–corn	21.23 ± 0.26^a,b,y^	20.20 ± 0.29^a,y,z^	19.67 ± 0.31^a,z^	trt	<0.0001
Rye–corn	21.69 ± 0.29^a,y^	19.98 ± 0.33^a,b,z^	20.00 ± 0.31^a,z^	line	<0.0001
Rye–rye	20.36 ± 0.29^a,b,z^	19.92 ± 0.29^a,z^	19.61 ± 0.31^a,z^	trt*line	0.0238
Corn–rye	19.96 ± 0.27^b,y^	18.59 ± 0.29^b,z^	19.78 ± 0.31^a,y,z^		

*Data are expressed as the LSmean ± SE*.

*^a–c^Significant differences between the treatments within the column at each time point*.

*^x–z^Significant difference between the genetic lines within the rows at each time point*.

#### Tibia Ash

At day 20, treatment, genetic line, and interaction between the two variables had a significant effect on tibia ash (*P* < 0.0001; Table 4). Modern and 1995 broilers fed corn in the second phase of the experiment had significantly higher tibia ash content than rye-fed chicks (*P* < 0.0001). In the Jungle Fowl, there was no significant difference in tibia ash between the treatments. Between genetic lines, modern broilers had significantly higher tibia ash than the 1995 broilers in the rye–rye (*P* < 0.0001) and corn–rye (*P* < 0.0001) groups. Similar results were observed in the Jungle Fowl where the rye–rye (*P* = 0.0083) and corn–rye (*P* = 0.0016) groups had significantly higher tibia ash than the 1995 broiler. Modern broiler in the rye–corn group had significantly higher tibia ash than the 1995 broiler (*P* = 0.0002) and the Jungle Fowl (*P* < 0.0001). In the corn–corn group, modern broilers had significantly higher ash content than the 1995 broilers (*P* = 0.0167) and the Jungle Fowl (*P* < 0.0001). As well, 1995 broiler had significantly higher tibia ash content than Jungle Fowl (*P* = 0.034).

#### Calcium and Phosphorus Content

Modern broilers in the corn–corn group had significantly higher tibia calcium content than those chicks in the rye–rye (*P* = 0.0152) and corn–rye (*P* < 0.0001). Similar results were observed in rye–corn groups, where the modern broilers in the rye–corn group had significantly higher tibia calcium content than the rye–rye (*P* = 0.0004) and the corn–rye (*P* < 0.0001) groups. Modern broilers in the rye–corn group had higher phosphorus content than the corn–rye group (*P* = 0.0036); however, no differences were observed between the other treatment groups. In the 1995 broilers, the corn–corn group had significantly higher tibia calcium concentrations than the corn–rye group (*P* = 0.003). The corn–rye group had significantly lower tibia phosphorus content than the corn–corn (*P* = 0.0064) and rye–rye (*P* = 0.0493) groups. There was no difference in calcium and phosphorus content between the other treatment groups in the 1995 broilers. There was no difference in calcium or phosphorus levels between treatments in the Jungle Fowl. Calcium content in the corn–corn group was significantly higher in the modern broiler compared with the 1995 broiler (*P* = 0.0045) and the Jungle Fowl (*P* = 0.0499). For the rye–corn group, modern broilers had significantly higher tibia calcium content than the Jungle Fowl (*P* = 0.004) and the 1995 broiler line (*P* = < 0.0001). In the corn–rye group, Jungle Fowl had significantly higher calcium content than the 1995 line (*P* = 0.0024). Tibia phosphorus content in the corn–corn group was significantly higher in the modern broiler than the Jungle Fowl (*P* = 0.016). In the rye–corn group, the modern broiler had significantly higher phosphorus content in the tibia than the 1995 broiler (*P* = 0.0166) or the Jungle Fowl (*P* = 0.0115). In the corn–rye group, modern broilers had significantly higher tibia phosphorus content than the 1995 broiler (*P* = 0.0287). There was no difference in calcium or phosphorus content between genetic lines for those birds maintained on the rye treatment throughout the experiment (Table 4).

### Morphometric Analysis

Table 5 shows the results of the evaluation of a nutritional rehabilitation model on morphometric analysis of the duodenum in three genetic chicken lines fed rye or corn at varying time points at day 20 of age.

**Table 5:** Evaluation of a nutritional rehabilitation model on morphometric analysis of duodenum in three genetic chicken lines fed rye or corn at varying time points at day 20 of age.

Treatment/variable	Genetic line			Variable	*P*-value
	Modern broiler	1995 Line	Jungle Fowl		
**Villus height (μm)**					
Corn–corn	242.48 ± 15.94	244.57 ± 14.76	190.32 ± 17.47	trt	0.0003
Rye–corn	276.67 ± 15.94	250.22 ± 15.94	227.3 ± 17.47	line	<0.0001
Rye–rye	303.81 ± 15.94	313.13 ± 17.46	205.74 ± 15.94	trt*line	0.059
Corn–rye	329.37 ± 15.94	324.17 ± 15.94	200.06 ± 19.53		
**Crypt depth (μm)**					
Corn–corn	30.47 ± 1.84^b,z^	24.41 ± 1.71^c,z^	22.77 ± 2.02^a,z^	trt	<0.0001
Rye–corn	29.69 ± 1.84^b,z^	29.70 ± 1.84^b,c,z^	24.18 ± 2.02^a,z^	line	<0.0001
Rye–rye	39.90 ± 1.84^a,y^	42.90 ± 2.02^a,y^	27.17 ± 1.84^a,z^	trt*line	0.0014
Corn–rye	41.64 ± 1.84^a,y^	37.63 ± 1.84^b,y^	23.08 ± 2.26^a,z^		
**Villus height to crypt depth (μm)**					
Corn–corn	7.96 ± 1.21	10.03 ± 1.12	8.56 ± 1.32	trt	0.9001
Rye–corn	9.57 ± 1.21	8.49 ± 1.21	9.44 ± 1.32	line	0.1661
Rye–rye	7.61 ± 1.21	7.38 ± 1.32	13.04 ± 1.21	trt*line	0.0886
Corn–rye	8.04 ± 1.21	8.94 ± 1.21	8.88 ± 1.48		
**Muscularis (μm)**					
Corn–corn	33.77 ± 2.87	33.58 ± 2.66	29.44 ± 3.15	trt	0.203
Rye–corn	30.12 ± 2.87	31.52 ± 2.87	24.70 ± 3.15	line	0.0391
Rye–rye	35.59 ± 2.87	29.59 ± 3.15	36.68 ± 2.87	trt*line	0.0609
Corn–rye	35.03 ± 2.87	37.63 ± 2.87	22.91 ± 3.52		

*Data are expressed as the LSmean ± SE*.

*^a–c^Significant differences between the treatments within the column at each time point*.

*^y–z^Significant difference between the genetic lines within the rows at each time point*.

#### Duodenum

Duodenal VH was significantly affected by treatment (*P* = 0.0003) and genetic line (*P* < 0.0001); however, there was no significant interaction between the two variables (*P* = 0.059). Regardless of genetic line, the corn–corn group had significantly shorter villi than the rye–rye (*P* = 0.0032) and the corn–rye groups (*P* = 0.0004). Overall, Jungle Fowl had significantly shorter villi than the modern broiler (*P* < 0.0001) and the 1995 broiler (*P* < 0.0001); however, VH was similar between the modern and 1995 broiler (*P* = 0.9009). Duodenal CD was altered by treatment, genetic line, and the interaction between the two variables had a significant effect on duodenal CD (*P* < 0.0014). Modern broilers in the corn–corn and rye–corn treatment group had significantly shorter CD than those fed rye–rye (*P* < 0.05) or corn–rye (*P* < 0.05). 1995 Chickens in the corn–corn group had significantly shorter CD than those chicks in the rye–rye (*P* < 0.0001) and the corn–rye (*P* = 0.0001). The 1995 chicks in the rye–corn group had significantly lower CD than the rye–rye (*P* = 0.0006) fed chicks; however, there was no differences in CD between the corn–rye and rye–corn groups (*P* = 0.1236). In the Jungle Fowl, there was no significant difference between treatments for duodenal VH and CD. There were no significant differences in CD between genetic lines in chicks fed a corn-based diet in the second phase of the experiment. However, Jungle Fowl in the rye–rye and corn–rye group had significantly shorter CD than the modern broiler (*P* < 0.005) and 1995 broilers (*P* < 0.005). In the duodenum, there was no difference between treatments, genetic lines, or interaction between the treatment and genetic lines in the VH:CD (Table 5). Genetic line had a significant effect on muscularis thickness (*P* = 0.0391) where modern broiler had a significantly thicker muscularis than the Jungle Fowl (*P* = 0.0480). Treatment (*P* = 0.203) and interaction between the treatments and genetic lines were similar for muscularis thickness (*P* = 0.0609).

#### Ileum

The results of the evaluation of a nutritional rehabilitation model on morphometric analysis of the ileum in three genetic chicken lines are summarized in Table 6. Unlike the duodenum, the ileum was relative unaltered by line, dietary treatment, or the interaction of line by dietary treatment. A significant interaction was observed between the treatments and genetic line for ileal VH (*P* = 0.048); however, after multiple testing correction (Tukey multiple comparison test), no statistical differences were observed between treatments or genetic lines for ileal VH. Jungle Fowl villi were shorter than the modern broiler (*P* = 0.0024) and the 1995 broiler (*P* = 0.0301). Genetic line had a significant effect on VH, CD, and muscularis thickness (*P* < 0.01) where modern broilers had significantly taller villi, deeper CDs, and thicker muscularis compared to the 1995 broiler (*P* < 0.05) and Jungle Fowl (*P* < 0.05). The 1995 broilers was in intermediate between Jungle Fowl and the modern broiler for all three traits (*P* = 0.0003).

**Table 6:** Evaluation of a nutritional rehabilitation model on morphometric analysis of ileum in three genetic chicken lines fed rye or corn at varying time points at day 20 of age.

Treatment/variable	Genetic line			Variable	*P*-value
	Modern broiler	1995 Line	Jungle Fowl		
**Villus height (μm)**					
Corn–corn	150.90 ± 11.49	181.8 ± 10.64	136.10 ± 12.59	trt	0.3338
Rye–corn	193.99 ± 11.49	171.58 ± 11.49	156.2 ± 12.59	line	0.0026
Rye–rye	165.61 ± 11.49	164.28 ± 11.49	142.55 ± 11.49	trt*line	0.048
Corn–rye	190.36 ± 11.49	144.07 ± 11.49	141.56 ± 11.49		
**Crypt depth (μm)**					
Corn–corn	25.84 ± 1.79	26.78 ± 1.66	17.61 ± 1.96	trt	0.1083
Rye–corn	30.30 ± 1.79	26.36 ± 1.78	22.04 ± 1.96	line	<0.0001
Rye–rye	28.46 ± 1.79	24.75 ± 1.78	19.92 ± 1.79	trt*line	0.2953
Corn–rye	28.961 ± 1.79	22.40 ± 1.78	19.91 ± 1.79		
**Villus height to crypt depth (μm)**					
Corn–corn	6.62 ± 0.39	8.66 ± 0.36	6.92 ± 0.43	trt	<0.0001
Rye–corn	7.62 ± 0.39	7.54 ± 0.39	6.36 ± 0.43	line	0.8452
Rye–rye	4.52 ± 0.39	5.05 ± 0.39	5.30 ± 0.39	trt*line	0.4854
Corn–rye	4.54 ± 0.39	4.75 ± 0.39	5.99 ± 0.39		
**Muscularis (μm)**					
Corn–corn	26.34 ± 2.05	26.39 ± 1.89	25.11 ± 2.24	trt	0.3216
Rye–corn	29.80 ± 2.05	26.50 ± 2.04	26.61 ± 2.24	line	0.0112
Rye–rye	28.67 ± 2.05	25.03 ± 2.04	25.03 ± 2.04	trt*line	0.0561
Corn–rye	35.53 ± 2.05	25.65 ± 2.04	22.84 ± 2.04		

*Data are expressed as the LSmean ± SE*.

*No differences were detected after conducted Tukey’s post hoc test*.

Dietary treatment had a significant effect on VH:CD ration, where the corn–corn group had a significant higher VH:CD ratio than the rye–rye (*P* = 0.0002) and corn–rye (*P* = 0.0003) groups. Similar results were observed in the rye–corn group, where the rye–rye (*P* = 0.0292) and the corn–rye (*P* = 0.0467) groups had a significantly lower VH:CD ration. All other effects were not altered and *P*-values can be found in Table 6.

## Discussion

### BW/Compensatory Gain

Chickens may be the ideal animal for preclinical studies of growth because of their rapid growth rates and the extensive amount of literature on poultry nutrition (21, 23, 36, 37, 50). It is evident in this study that compensatory gain occurred in the modern broilers as the rye–corn treatment group had the same BW and BWG as those maintained a corn-based diet throughout the experiment. Interestingly, in the 1995 broilers, the rye–corn treatment group exhibited compensatory growth as they had a higher BW and BWG than those chicks in the corn–rye group. However, 1995 broilers in the corn–corn treatment group weighed significantly more than the rye–corn group suggesting there was not a full recovery within the observed timeframe. The slower compensatory growth rate observed in the 1995 broilers was similar to that of stunted children in developing countries, where the effects of stunting are often permanent after 3 years of age (51). Therefore, the 1995 broilers may be the most appropriate model organism when determining clinical interventions. The difference in BW and BWG between the modern broiler and the 1995 broiler in the rye–corn group also suggests that genetic selection within the last 20 years has allowed modern broilers to exhibit compensatory gain after a period of undernutrition. Similar results were observed by Zubair and Leeson (29) who reported that re-feeding broilers after quantitative or qualitative feed restriction allowed for some BW to be recovered but BW was not fully compensated (29). Interestingly, this article was published in 1996, so potentially these birds may have had a similar genetic profile to the 1995 line used in this trial (29). In the Jungle Fowl, there were no significant differences in BW between the treatments at day 10 or day 20. When Jungle Fowl were placed on a choice fed diet, they choose nutrients to support optimum growth (32), suggesting that Jungle Fowl are not adapted to a particular diet and they eat to their metabolic requirements. Since both the rye-based diet and corn-based diet were isonitrogenous, perhaps both the diets contained the protein levels to meet maintenance and growth requirements of Jungle Fowl. Furthermore, similar to Leghorn chickens, Jungle Fowl eat until their energy and protein requirements are met (32). By contrast, broilers eat until the gut capacity is full (52). However, Jungle Fowl in the corn–corn group had significantly higher BWG than the rye–rye group, suggesting that the anti-nutritional factors of a rye-based diet also affected these chickens. As far as we are aware, this is the first study performed looking at the effect on high NSP diets on compensatory gain in chickens. Previous research used quantitative or qualitative feed restriction to study compensatory gain (53, 54). Rosebrough and McMurtry (55) placed broilers on short-term energy restriction from 6 to 12 days of age and the allowed *ad libitum* access feed, which led to compensatory gain. Zhan et al. (31) reported that the average daily gain was significantly lower when chickens were feed restricted for 4 h from 1 to 21 days of age. However, once the chicks had *ad libitum* access to feed there was no significant difference in BW or average daily gain, attributing the compensatory gain to the reduction in basal metabolic rate by reducing the amount of thyroid hormone (31). In addition, BW and BWG were highest in modern broilers, followed by 1995 chickens, and Jungle Fowl had the lowest BW. These results are in agreement with Wall and Anthony reporting that it takes 93 days for Jungle Fowl chickens to reach the same BW of broiler breeders (38). Genetic selection for various production traits has reduced genetic diversity in broilers and laying hens (56). This may be a contributing factor as to why the broilers in this study were more affected by the anti-nutritional factors in a rye-based diet when compared with the Jungle Fowl.

From the present trial, it is evident that chicks maintained on rye-based diet throughout had significantly lower BW and BWG in modern and 1995 broilers especially when compared with Jungle Fowl. This is because whole rye contains of a high amount of NSP, such as β-glucan and arabinoxylans. These soluble NSP absorb water and increasing digesta viscosity which impairs nutrient digestion and absorption. The lack of nutrient digestion and absorption in the small intestine allows more nutrients to enter the lower intestine, providing bacteria a nutrient source and leading to dysbiosis within the gut. Hence, rye diets evoke mucosal damage in chickens that alter digesta viscosity, increase leakage throughout the intestinal tract, and affect the microbiota composition and bone mineralization (15, 16). Studies published by our laboratories have shown that rye-based diets significantly increased both viscosity and *Clostridium perfringens* and *Clostridium difficile* proliferation when compared with corn-based diets (9). Since poultry has little or no intrinsic enzymes capable of hydrolyzing these NSP, exogenous carbohydrases are used as additives to reduce the negative impact of these anti-nutritive factors. Many of the clinical and pathological effects reported in poultry consuming diets high in NSP are also observed in stunted children living in low-income countries (13, 14). These include stunting, development of enteropathy, alteration in gut microbiome profile, bacterial translocation, reduction in nutrient digestion and absorption, as well as poor bone mineralization (4, 10–14, 17). This suggests that a rye-based diet is a viable approach to induce undernutrition in chickens to study compensatory growth and clinical interventions in malnourished children.

### Bone Health

The increase intestinal viscosity caused by high NSP diets impairs digestion and absorption of nutrients (57). Furthermore, the high concentration of phytate in rye has been linked to contribute to the poor bone mineralization (58, 59). Nevertheless, supplementation of enzymes or DFM that produce exogenous enzymes ameliorate these negatives effects (41, 60, 61). Modern broilers fed corn in the second phase of the experiment had stronger tibias, higher amounts of tibia ash, and higher levels of calcium than broilers fed rye. This indicates that chicks in the rye–corn group were able to recover bone parameters within the observed time frame. The 1995 broilers fed only corn had significantly stronger tibias than any of the other treatment groups including the rye–corn group. However, 1995 broilers in the rye–corn group had stronger tibias than those birds fed rye in the second phase of the experiment indicating some recovery in bone strength has occurred. Tibia bone ash improved when a low phosphorus diets were supplemented with phytase, suggesting that the enzyme increased mineral availability (62). Therefore, it likely that switching from a rye to a corn-based diet increased mineral availability allowing for improved bone quality in modern and 1995 broilers. Modern broilers fed corn had significantly more tibia ash and calcium than the 1995 broiler and Jungle Fowl. This further supports the idea the modern broilers can recover from insult faster and may even be more resistant to the anti-nutritional factors in a high NSP diet. Similarly, Jungle Fowl chickens, fed in the corn–corn group had significantly stronger tibias than those fed rye at any phase of the experiment. This finding did not correlate with the performance data where there were no differences in BW or BWG between treatments. This suggests that Jungle Fowl chickens consuming a rye-based diet at any phase of the experiment may be meeting energy requirements for growth, but mineral deficiencies may be affecting bone strength. However, there was no difference between treatments for percent tibia ash, or calcium and phosphorus levels.

### Histology

Selection for growth has altered the morphology of the gastrointestinal tract to accommodate nutrient absorption for rapid growth (63). In this experiment, there were no significant interaction between dietary treatments and genetics for VH, VH:CD, and muscularis in the duodenum. These results are similar to previous studies showing that wheat diets increased duodenal digesta viscosity but had no effect on morphology of the gastro intestinal tract (64, 65). In the duodenum, the chicks fed corn in the second phase of the experiment had statistically lower villi than rye-fed chicks. Previous research has found that chickens consuming diets high in fiber increase epithelial turnover with the consequent increase in VH, as a measure to try to compensate for the poor digestibility (33, 66, 67). Other studies have demonstrated that removing various section of the small intestine had little effect on BW, nitrogen retention, dry matter digestibility, due to hypertrophy of intestinal villi and epithelial cells in the remaining small intestine (68). Therefore, chickens fed rye in the second phase of the experiment may be trying to acquire more nutrients by increasing VH in an attempt to increase nutrient intake.

Crypt depth is an indicator of cell turnover rate, and shorter villi and deeper crypts are often indicators of toxin present in the lumen of the gastrointestinal tract (69, 70). The more energy required for cellular replication, the less is available for growth (70). Modern broilers fed corn in the second phase of the experiment had significantly shallower CD in the duodenum than those chicks fed rye in the second phase of the experiment. These data follow a similar trend to that of the BW and bone data, where chicks in the rye–corn group can recover and decrease CD after consumption of a rye-based diet. Previous work conducted in our laboratory suggest that the dysbacteriosis and high number of coliforms associated with rye diets in poultry is a source of toxins that not only induce intestinal inflammation and gut permeability but also deeper crypts (15, 16). In the duodenum, rye-fed broilers had deeper crypt but there was no significant difference in VH between treatments. Therefore, the intestinal epithelial cells have a high turnover rate but the same VH. Similar results have been reported where diets containing soluble NSP resulted in deeper crypts by shorter villi in the ileum (71). The short villi may be due to increased apoptosis when consuming a diet high in soluble NSP (72). Overall musculairs thickness in the duodenum was significantly higher in the modern broilers when compared with Jungle Fowl; however, there was no difference in muscularis thickness between dietary treatments. This may indicate that modern broilers have more intestinal inflammation of the muscularis. Recent studies in broiler chickens fed with a rye-/wheat-based diet had T-cell aggregates in the mucosa as an indicator of excessive immune stimulation as well as a thinner tunica muscularis when compared with those fed a maize-based diet, suggesting that excessive stimulation of the immune system led to thin muscularis, which may explain why there were no differences in muscularis thickness treatment groups (72).

There were no significant interaction between dietary treatments and genetics for CD, VH:CD, and muscularis in the duodenum. The VH:CD ratio was significantly higher in corn-fed chicks when compared with rye-fed chicks in the ileum. Comparable results were observed by Mathlouthi et al. (73) for the VH:CD ratio in corn-fed chickens had significantly higher ratio than the rye-fed birds, but the addition of enzymes improved VH to that of the corn-fed birds (73). The authors hypothesized that supplementation of enzymes reduced digest viscosity which led to longer villus, hence increasing broiler performance (73). In our study, we also observed that switching from a rye to a corn-based diet improved VH:CD ratio. However, in this experiment, the improvement was not due to increase VH but a decrease in CD. Although digesta viscosity was not recorded in the current experiment, previous research published by our laboratory using the same diet formulation reported high digesta viscosity in a rye-based diet (15, 16).

Regardless of dietary treatment, modern broilers fed rye in any phase of the experiment had significantly deeper CD, longer villi, and thicker muscularis than the Jungle Fowl in both the duodenum and ileum. Zulkifli et al. (33) attributed the difference in intestinal size and surface area to the different growth rates (33). Although we did not measure intestinal length, Wall and Anthony (38) found that broiler breeds had heavier and longer digestive tracts than the Jungle Fowl, suggesting that selection for feed efficiency affects total size of the gastrointestinal tract. It has also been reported that is a positive correlation for growth rate and digestion and absorption of nutrient (74).

## Conclusion

It is evident from this study that the consumption of a rye-based diet had malabsorptive effects on broilers, especially the in the 1995 broiler in the rye–corn group as they were unable to fully recover BW and tibia strength. The results of this study suggest that chickens are a viable model to study nutritional rehabilitation in human trials. Specifically, the 1995 chicken line exhibited a compensatory gain patterns to that observed in malnourished children. Our next objective is to design a nutritional trial evaluating our DFM spore base probiotic (that produce exogenous cellulase, xylanase, amilase, β-galactase, phytase, proteases, and lipases) alone or in combination with selective lactic acid-based probiotic, using this model in 1995 chickens, before we expand our trials in children to test the lessons learned from our preclinical model. This process would include identifying a site in sub-Saharan Africa where these interventions could be tested in children with EE, ensuring that all of the human subject’s protections are in place, and developing the best strategies for measuring clinical outcomes.

## Ethics Statement

All animal procedures were approved and in compliance with Institutional Animal Care and Use Committee (IACUC) at the University of Arkansas, Fayetteville (protocol #15006).

## Author Contributions

MB, JL, and GT contributed to the overall study design and supervised all research. DK, SD, EG, SB, NA, and RM-G carried out the experiments and acquisition of data. MB, JK, RM-G, XH-V, and GT drafted and revised the first version of the manuscript. JL and GT analyzed the data. NA, WB, BH, and GT drafted the article and revised it critically for important intellectual content. XH-V and GT were responsible for the final editing of the manuscript. All the authors reviewed and finally approved the manuscript.

## Conflict of Interest Statement

The authors declare that the research was conducted in the absence of any commercial or financial relationships that could be construed as a potential conflict of interest. The reviewer GC and handling editor declared their shared affiliation.
